# Experimental Models of Foamy Macrophages and Approaches for Dissecting the Mechanisms of Lipid Accumulation and Consumption during Dormancy and Reactivation of Tuberculosis

**DOI:** 10.3389/fcimb.2016.00122

**Published:** 2016-10-07

**Authors:** Pierre Santucci, Feriel Bouzid, Nabil Smichi, Isabelle Poncin, Laurent Kremer, Chantal De Chastellier, Michel Drancourt, Stéphane Canaan

**Affiliations:** ^1^Aix-Marseille Université, Centre National de la Recherche Scientifique, EIPLMarseille, France; ^2^Aix-Marseille Université, Institut National de la Santé et de la Recherche Médicale, Centre National de la Recherche Scientifique, Institut de Recherche pour le Développement, URMITEMarseille, France; ^3^Centre d'études d'agents Pathogènes et Biotechnologies pour la Santé, Centre National de la Recherche Scientifique FRE3689, Université de MontpellierMontpellier, France; ^4^Centre d'études d'agents Pathogènes et Biotechnologies pour la Santé, Institut National de la Santé et de la Recherche MédicaleMontpellier, France

**Keywords:** granuloma, adipocyte, amoeba, mycobacteria, pathogenesis, lipid body

## Abstract

Despite a slight decline since 2014, tuberculosis (TB) remains the major deadly infectious disease worldwide with about 1.5 million deaths each year and with about one-third of the population being latently infected with *Mycobacterium tuberculosis*, the etiologic agent of TB. During primo-infection, the recruitment of immune cells leads to the formation of highly organized granulomas. Among the different cells, one outstanding subpopulation is the foamy macrophage (FM), characterized by the abundance of triacylglycerol-rich lipid bodies (LB). *M. tuberculosis* can reside in FM, where it acquires, from host LB, the neutral lipids which are subsequently processed and stored by the bacilli in the form of intracytosolic lipid inclusions (ILI). Although host LB can be viewed as a reservoir of nutrients for the pathogen during latency, the molecular mechanisms whereby intraphagosomal mycobacteria interact with LB and assimilate the LB-derived lipids are only beginning to be understood. Past studies have emphasized that these physiological processes are critical to the *M. tuberculosis* infectious-life cycle, for propagation of the infection, establishment of the dormancy state and reactivation of the disease. In recent years, several animal and cellular models have been developed with the aim of dissecting these complex processes and of determining the nature and contribution of their key players. Herein, we review some of the *in vitro* and *in vivo* models which allowed to gain significant insight into lipid accumulation and consumption in *M. tuberculosis*, two important events that are directly linked to pathogenicity, granuloma formation/maintenance and survival of the tubercle bacillus under non-replicative conditions. We also discuss the advantages and limitations of each model, hoping that this will serve as a guide for future investigations dedicated to persistence and innovative therapeutic approaches against TB.

## Introduction

With 9.6 million new cases and 1.5 million deaths in 2014, tuberculosis (TB) ranks alongside with HIV as the major cause of deadly infectious diseases (WHO, 2015). It is estimated that about one-third of the world population is infected with the etiological agent of TB, *Mycobacterium tuberculosis*, but only 5 to 10% of the infected individuals are at risk of developing active disease within a few years. Most individuals remain asymptomatic after the primary infection. In such individuals, bacilli do not divide but may persist for decades in a dormant state until reactivation of bacilli leads to active disease (WHO, 2015).

Infection with *M. tuberculosis* follows a relatively well-defined sequence of events (Russell, 2001; Russell et al., 2009a). After inhalation of contaminated aerosols, *M. tuberculosis* reaches the lungs, where the bacilli are phagocytosed by alveolar macrophages. Prognosis of the disease, viz, active or dormant, will depend on the host's ability to contain the bacilli at the site of infection. In most cases, the bacilli induce a local pro-inflammatory response which leads to the recruitment of other immune cells (macrophages, dendritic cells and lymphocytes) from neighboring blood vessels. The latter accumulate around the alveolar macrophages, at the site of infection, to form a granuloma which is the hallmark of TB (Russell, 2007; Peyron et al., 2008; Gideon and Flynn, 2011; Galagan, 2014).

The tuberculous granuloma is a highly elaborate host protective structure, which represents an essential immune mechanism for containing the bacilli inside the lungs without fully eradicating them (Kaufmann, 2001; Russell, 2001). The formation process, the composition and maintenance of this well-organized system have been extensively investigated and reviewed over the last decade (Russell et al., 2009a; Ramakrishnan, 2012; Guirado and Schlesinger, 2013). Briefly, macrophages differentiate into epithelioid cells or become highly vacuolated or even fuse to form multi-nucleated giant cells (Puissegur et al., 2007; Russell et al., 2009a; Feng et al., 2014). Other macrophages, which are the focus of intense research, differentiate into foam cells characterized by the intracytosolic accumulation of neutral lipids in the form of lipid bodies (LB) also known as lipid droplets or lipid vacuoles (Daniel et al., 2004; Deb et al., 2006; Russell et al., 2009a). These specific foamy macrophages (FM) are found in the interface region flanking the central necrotic center of the granuloma (Peyron et al., 2008).

Traditionally, FM were qualified as passive organelles involved in lipid storage. More recently, it became clear that these organelles play a central role in several inflammatory diseases, such as atherosclerosis, or in chronic infectious diseases (Russell et al., 2009b). Different myeloid cell types have been used to generate lipid-loaded foam cells and to provide insights into their contribution in chronic inflammatory conditions. In the context of TB, pathologists have described the presence of FM within granulomatous structures in both experimentally-infected animals and patients (Ridley and Ridley, 1987; Cardona et al., 2000), especially in individuals developing secondary TB (Pagel, 1925; Florey, 1958; Hunter et al., 2007), thereby suggesting that FM may play a central role in mycobacterial persistence and reactivation.

Since *M. tuberculosis* is facing important physiological changes as TB progresses toward containment of the bacilli within granulomas, it is assumed that this involves adaptive requirements that are distinct from those encountered during the initial phase of infection (Hingley-Wilson et al., 2003; Mckinney and Gomez, 2003; Sassetti and Rubin, 2003). Among these, accumulation of neutral lipids, essentially triacylglycerol (TAG), within intracytosolic lipid inclusions (ILI) (Dhouib et al., 2011), has been proposed to serve as a source of carbon and energy, and represents a hallmark of persistent and non-dividing mycobacteria (Garton et al., 2002; Daniel et al., 2011). Therefore, residing within specialized lipid-rich cells, rather than in conventional macrophages, would be beneficial and even promote *M. tuberculosis* persistence within granulomatous lesions. This also implies that bacilli must produce lipolytic enzymes and/or activate host hydrolytic enzymes in order to degrade the host neutral lipids into free fatty acids (FFA) and then re-synthesize TAG for subsequent ILI formation. Clearly, the abundance of genes encoding enzymes involved in lipid metabolism (Cole et al., 1998) strongly suggests that *M. tuberculosis* uses host-derived lipids while persisting *in vivo* (Höner Zu Bentrup and Russell, 2001).

The purpose of the present review is firstly to recall how host cells become foamy, by accumulating neutral lipids in the form of LB and, second, to describe and compare different foam cell models and approaches that have served to delineate lipid accumulation and consumption processes developed by *M. tuberculosis* and other mycobacteria within their host cells. Advantages and drawbacks of each model are also discussed.

## Lipid bodies in foam cells: a reservoir of lipids for persistent *M. tuberculosis*

**Figure 1:**
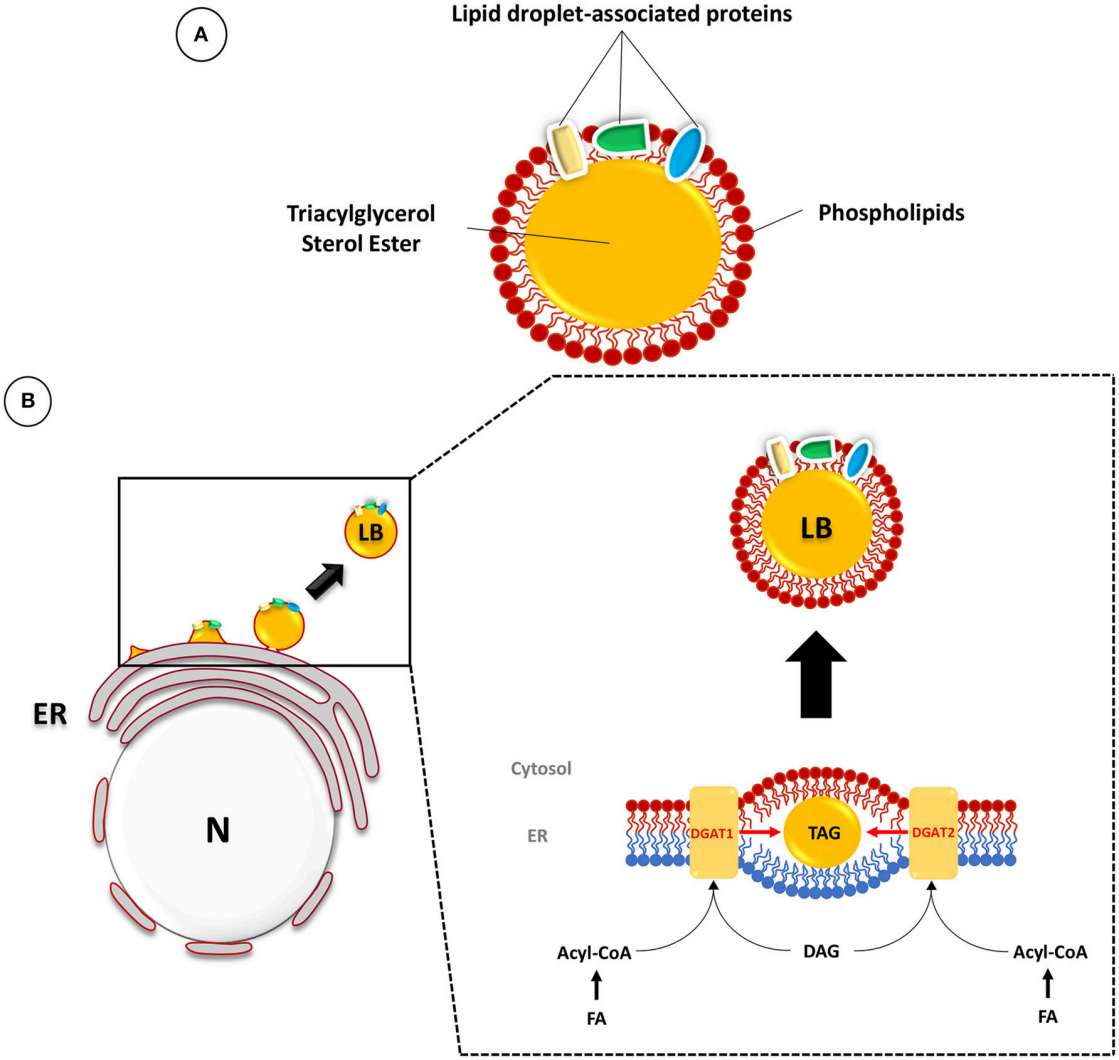
**Composition and biogenesis of lipid bodies (LB) in mammalian cells. (A)** The LB is composed of a core of TAG and sterol esters surrounded by a phospholipid monolayer and associated proteins. **(B)** TAG synthesis occurs between the two membrane leaflets of the endoplasmic reticulum (ER) (inner leaflet in blue and outer leaflet in red). The prevailing model suggests that host LB originate at the ER, where fatty acids (FA) are resynthesized to form triacylglycerol (TAG) and sterol esters. These compounds accumulate in the hydrophobic space between the two leaflets (in red: outer leaflet; in blue: inner leaflet) of the ER bilayer membrane. The final step is catalyzed by the diacylglycerol acyltransferases DGAT1 and DGAT2. The conversion of diacylglycerol (DAG) and Acyl-CoA into TAG leads to the formation of a lipid globule which will expand dynamically and finally be released into the cytosol as a mature LB surrounded by phospholipids of the cytosolic leaflet of the ER bilayer membrane. Adapted from (Murphy, 2001; Kassan et al., 2013; Pol et al., 2014).

The presence of neutral lipid storage organelles in the cytoplasm of most eukaryotes (named LB) and prokaryotes (named ILI) has been known for a long time. In all cases, these ubiquitous structures are composed of a core of neutral lipids, such as sterol esters and TAG, surrounded by a monolayer of phospholipids, in which cholesterol ester and proteins are inserted (Figure 1A; Murphy, 2001; Martin and Parton, 2005; Pol et al., 2014). A large number of associated proteins have been discovered in recent studies devoted to the composition and dynamics of LB formation (Brasaemle et al., 2004; Yang et al., 2012). Furthermore, it is now clear that, beyond neutral lipid storage, the functions of LB are multiple, and this knowledge has given rise to new hypotheses concerning the role of these organelles in several physiological processes (Saka and Valdivia, 2012).

The current models of LB biogenesis have been reviewed extensively (Murphy and Vance, 1999; Ohsaki et al., 2009; Saka and Valdivia, 2012). The prevailing model suggests that host LB originate at the endoplasmic reticulum (ER), where fatty acids are used to re-synthesize TAG and sterol esters. The *de novo* anabolic process leading to TAG synthesis relies on multiple enzymatic steps, the final one being catalyzed by diacylglycerol acyltransferases (DGAT) (Harris et al., 2011; Pol et al., 2014). Among these, DGAT1 and DGAT2 are located in the ER where they can synthesize TAG from diacylglycerol (DAG) and acyl-CoA. TAG and sterol esters then accumulate in the hydrophobic space between the leaflets of the ER bilayer membrane, where they are subsequently engulfed by phospholipids of the cytosolic leaflet and pinched off the ER membrane into the cytosol (Figure 1B; Murphy, 2001; Kassan et al., 2013; Pol et al., 2014).

Over one decade ago, Garton et al. established that *M. tuberculosis* could accumulate neutral lipids in the form of ILI *in vitro* and in sputum of TB patients (Garton et al., 2002). Since then, ILI formation has been extensively described and analyzed in several mycobacterial species, including *M. tuberculosis* (Mckinney et al., 2000; Daniel et al., 2004, 2011; Deb et al., 2006), *M. bovis* BCG (Low et al., 2009, 2010), *M. leprae* (Mattos et al., 2010, 2011), *M. abscessus* (Viljoen et al., 2016), and *M. smegmatis* (Dhouib et al., 2011). It has been proposed, but not yet proven, that storage of fatty acids in the form of TAG within ILI could serve as a source of carbon and energy during dormancy and reactivation of *M. tuberculosis* (Peyron et al., 2008; Low et al., 2009; Daniel et al., 2011; Dhouib et al., 2011).

Currently, almost nothing is known concerning the biological mechanism by which the storage of fatty acids may promote mycobacterial intracellular survival. Several hypotheses have been raised over the years, suggesting that ILI do not only serve as carbon source during infection, but could also reduce a wide range of oxido-reductive and metabolic stress (Kumar et al., 2011; Lee et al., 2013) It has also been proposed that the TAG accumulation can function in metabolic coupling to balance changes in metabolism of other *M. tuberculosis* lipids (Jain et al., 2007).

Accumulation of neutral lipids in the form of ILI has also been demonstrated *in vitro* or *in vivo* for a wide range of micro-organisms including Rhodoccoccus, Hepatitis C, and Dengue viruses, or Chlamydia (Saka and Valdivia, 2012; Alvarez, 2016) where they may serve other purposes.

Stimulated by these observations, several investigators have focused their activity on the search of external stimuli that may promote the accumulation of LB in macrophages and in other cells, with the aim of addressing the following key questions: (i) How do mycobacteria gain access to lipids from FM? (ii) How do they translocate the cellular lipids into their own cytoplasm? (iii) How are ILI formed and what is their composition? (iv) What are the lipolytic enzymes (from the host and mycobacteria) involved in these mechanisms? and (v) Is the arrest/resumption of mycobacterial division occurring during persistence and reactivation, respectively, linked to LB and ILI formation/consumption? At least some of these questions have been addressed thanks to the recent development of a panel of experimental models.

## Experimental models and approaches for studying mycobacterial lipid metabolism during the dormancy and reactivation stages of TB

Several stimuli were tested for designing foam cell models that rest upon the following observations. Firstly, the granuloma is considered to be a hypoxic environment. The high cell density and poor vascularization seems to be responsible for this low oxygen content which has been shown to promote foam cell formation (Bostrom et al., 2006; Cardoso et al., 2015; Datta et al., 2016). Secondly, most FM are localized in the close vicinity of the caseous center (Cáceres et al., 2009) where decaying cells may provide neutral lipids or phospholipids (Kim et al., 2010). Such external nutrients are known to play a major role on macrophage lipid metabolism (Shashkin et al., 2005) and may lead to a foamy phenotype *in vivo*. Finally, specific cell wall-associated components, such as mycolic acids from pathogenic mycobacteria, might be involved in the formation of lipid-loaded cells inside the granuloma (Peyron et al., 2008; Russell et al., 2009a).

### Animal models

Several models have been developed in mice to analyze different aspects of granuloma formation and the interactions between *M. tuberculosis* and the granulomatous environment. Karakousis et al. (2004) induced artificial granuloma formation in mice in order to mimic the caseous granuloma by physical containment of extracellular *M. tuberculosis*. This was achieved by inoculating liquid cultures of *M. tuberculosis* into the lumen of polyvinylidene fluoride (PVDF) hollow fibers with a syringe and a 20 gauge needle. The ends of the fibers were heat-sealed and individual fibers were prepared by heat-sealing at 2 cm intervals. Mice were anesthetized and the dorsal skin surface was sterilized with 70% ethanol. A small incision was made at the nape of the neck, and one fiber was deposited into the subcutaneous space of each flank. Incisions were closed with a surgical clip. The PVDF fibers have a molecular mass cut-off of 500 kDa, which allows diffusion of small soluble molecules but prevents the entry of host immune cells and the exit of bacilli. This model is particularly suitable for studying the behavior of extracellular *M. tuberculosis* within the host. Granulomatous lesions developed around the hollow fibers. In this micro-environment, the bacilli demonstrated an altered physiological state reminiscent of that of persistent bacilli which are characterized by stationary-state CFU counts, decreased metabolic activity and antimicrobial susceptibility. The accumulation of neutral lipids in the form of ILI within these bacilli was, however, not analyzed.

Another mouse model was developed by Geisel et al. (2005) and Rhoades et al. (2005) based on polystyrene beads coated with trehalose dimycolate (TDM) from *M. tuberculosis*. Following subcutaneous injection in mice of the TDM-loaded beads in a Matrigel matrix, the murine cells started to invade the matrix and remodeled the site under the influence of the mycobacterial lipid. Histological examination of the tissue sections revealed the abundance of adipophilin (ADRP or Plin2)-expressing foam cells around the inoculated beads and cryosections of TDM-induced granulomas showed an abundance of LB in foam cells (Kim et al., 2010).

One of the hallmarks of human TB is the development of granulomatous inflammatory lesions with central caseous necrosis and encapsulation of lesions by a fibrous connective tissue. In order to study interactions that occurs into granulomatous lesions, infection mouse models have been established allowing researchers to obtain crucial information concerning macrophages immune status, such as polarization, proliferation but also focusing onto cells differentiation during mycobacterial infection (Ordway et al., 2005, 2006). Based on that, few signaling networks have been proven to be associated to pathogen recognition response, macrophages immune and metabolic reprogramming, such as lipid metabolism and foamy appearance (Ordway et al., 2005; Schaale et al., 2013).

However, most commonly used mouse strains, such as BALB/c and C57BL/6, fail to develop well-organized granulomas with central necrosis (Kramnik et al., 2000; Keller et al., 2004). This prompted Kramnik and collaborators to developed another mouse strain, C3HeB/FeJ, referred to as the “Kramnik mouse model” (Kramnik et al., 1998) which, following infection with *M. tuberculosis*, develops highly organized and encapsulated necrotic lesions in the lungs resembling human pulmonary lesions (Pichugin et al., 2009). C3HeB/FeJ mice are highly susceptible to *M. tuberculosis* infection but possess an otherwise an intact and functional immune system. By combining immunohistochemical staining approaches with advanced imaging techniques, Driver et al. were able to follow disease progression in real time and localize bacilli and responsiveness to monotherapy within several days after exposure to low dose aerosol (Driver et al., 2012). More recently, Irwin et al. identified three distinct lesion types, arbitrarily called type I, II, and III, that vary in cell composition and organization in the granulomas resulting from aerosol infection (Irwin et al., 2015). Type I lesions most closely resembled classical human TB granulomas in that they are solid, encapsulated and caseous necrotic. They contain also large amounts of FM, of major interest for studying lipid accumulation and consumption during the persistence and reactivation phases of TB. However, to our knowledge, the “Kramnik mouse model” has not been used yet for such studies.

This model presents major advantages over other mouse models as the lesions, upon aerosol infection with *M. tuberculosis*, most closely resemble classical human TB granulomas. This model is a real asset for studying the progress of infection because the lesions contain all the immune cells involved in granuloma formation. In this context, *in situ* EM analysis of granulomas during the differentiation of infected macrophages into FM would be quite informative in terms of transfer to and accumulation of host cell neutral lipids in the form of ILI within the bacilli. These animal models are certainly attractive tools for *in vivo* drug assessments and monitoring the effect of drugs that can penetrate *M. tuberculosis*-containing granulomas. However, the complexity of the granulomatous lesions causes some experimental limitations. For instance, it is difficult to obtain homogenous preparations of pure FM or to isolate, in sufficient amounts, either FM or persistent bacilli confined within these cells, to undertake thorough studies to monitor physiological events, including lipid accumulation/consumption.

### *In vitro* model of human mycobacterial granulomas

This model was first developed by Puissegur et al. to decipher the cellular and molecular events corresponding to the very first steps of granuloma formation in the *M. tuberculosis*-infected host (Puissegur et al., 2004). Peripheral blood mononuclear cells (PBMC), isolated from the blood of healthy donors, were exposed to either live *M. bovis* BCG or to mycobacterial antigen-coated sepharose beads within a cell culture dish. This led to the progressive recruitment of macrophages around the live bacilli or coated beads which were then phagocytosed by macrophages. This event was followed by the differentiation of macrophages into multi-nucleated giant cells and epithelioid cells and finally, the recruitment of a ring of activated lymphocytes around the newly-formed structure. All in all, the formation and spatial organization of this *in vitro*-grown structure mimicked that of natural mycobacterial granulomas found in *M. tuberculosis*-infected patients.

This model was subsequently used by Peyron et al. to analyze the formation of FM and to study their interaction with *M. tuberculosi*s or *M. smegmatis* (Peyron et al., 2008). In this context, 1 × 10^6^ PBMC freshly isolated from healthy donors were purified by Ficoll density gradient separation and exposed to very low concentrations of viable *M. tuberculosis* (1 × 10^4^) or *M. smegmatis* (1 × 10^3^). The culture medium was RPMI-1640 + Glutamax containing 7.5% human AB serum. Three different imaging approaches were used to study LB formation. Briefly, for light microscopy studies, granuloma cells were collected and plated onto glass coverslips with a cytospin, fixed and stained with Oil red-O. After counterstaining with haematoxilin, cells were observed under an inverted microscope. For fluorescence analyses, granuloma cells were collected in PBS and LB were stained with Nile Red and observed under a confocal microscope. For electron microscopy (EM) studies, whole granulomas were fixed and processed for EM. Thin sections were prepared at different depths through the granulomas. The combination of these approaches at selected time points during granuloma formation allowed to visualize the morphological appearance of the different cell types, the presence or absence of bacilli within the different cells and the interaction between the bacilli and LB within FM. Of major interest was the differentiation of about 50% of the population of macrophages into FM within the *in vitro* granulomas obtained by exposure to *M. tuberculosis* but not to *M. smegmatis*. Within FM, as opposed to non-FM, bacilli were unable to divide. EM analyses of FM showed that LB, initially distributed randomly throughout the host cytosol, migrated toward *M. tuberculosis*-containing phagosomes with which they progressively made contact. Ultimately, the bacilli were enclosed in a compartment filled with lipids in which they accumulated, in turn, host cell lipids in the form of ILI (Peyron et al., 2008). These results showed, for the first time, that bacilli could use host-derived TAG as a potential source of nutrients to persist within granuloma FM. The process by which LB interacted with, and delivered their contents to, phagosomes remained, however, unclear until the advent of other models (see section Cells Exposed to Lipoproteins below).

**Figure 2:**
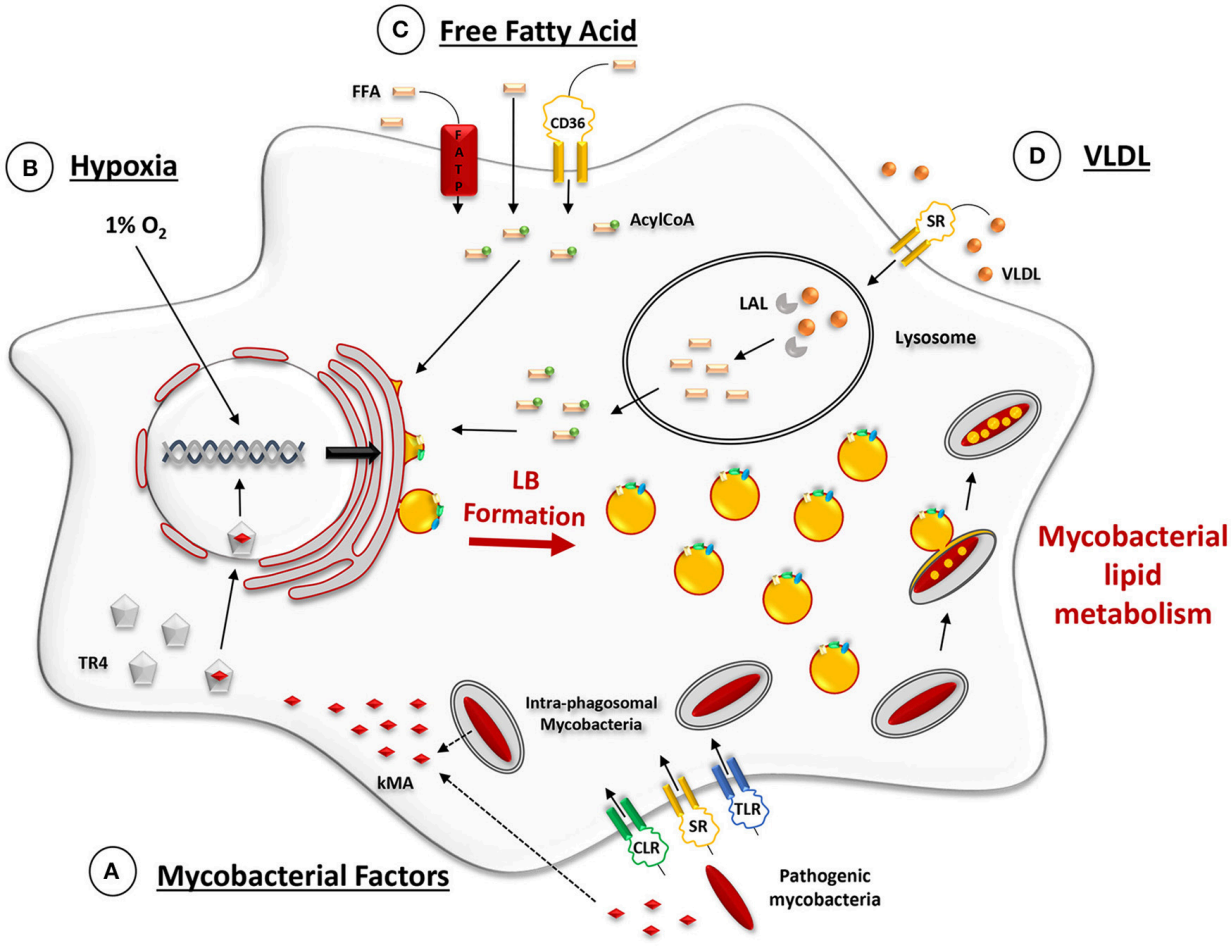
***Ex vivo*-induced mechanisms promoting LB formation in macrophage models. (A)** Phagocytic uptake of mycobacteria through scavenger (SR), C-type lectin (CLR), and Toll-like receptors (TLR). Lipid-containing moeities of the mycobacterial cell wall, such as keto-mycolic acids (kMA), are actively trafficked out of mycobacterium-containing phagosomes via fission vesicles (Beatty et al., 2000). At some point during or after vesicle trafficking through early and late endosomes, kMA, or their degradation products, are recognized by the testicular-receptor 4 (TR4) transcriptional factor which then translocates inside the nucleus and triggers a transcriptional response that promotes LB formation (Korf et al., 2005; Mahajan et al., 2012; Dkhar et al., 2014). **(B)** Cell culturing under conditions of hypoxia (1% O_2_) induces several transcriptional changes which act upon lipid metabolism and promote LB formation (Bostrom et al., 2006; Daniel et al., 2011). **(C)** Cell exposure to externally added free fatty acids (FFA). Internalization of FFA may occur *via three* main pathways: *(i)* diffusion through the plasma membrane, *(ii)* binding to the fatty acid transporter protein (FATP) or *(iii)* binding to SR, such as CD36. Once inside the cytosol, FFA are activated by an Acyl-CoA synthase and transported into the ER where LB are formed (Listenberger et al., 2003; Glatz et al., 2010). **(D)** Cell exposure to exogenous TAG-enriched VLDL. Lipids from the lipoproteins (VLDL) that are internalized by scavenger receptor-mediated endocytosis undergo hydrolysis in lysosomes by the lysosomal acid lipase (LAL). This provides the FFA for the subsequent building-up of TAG for LB formation, as mentioned above (Shashkin et al., 2005; Caire-Brändli et al., 2014).

Interestingly, this model allowed to identify mycobacterial factors involved in the differentiation of macrophages into FM within tuberculous granulomas (Figure 2A). Past work by Korf et al. had shown that mycolic acid (MA), a major lipid component of the cell wall of *M. tuberculosis*, administered to the peritoneal cavity or to the airways of mice could induce a unique macrophage morphotype, similar to the FM observed in human tuberculous granulomas (Korf et al., 2005). Following the above discovery, Peyron et al. compared FM formation in model granulomas obtained by exposure of PBMC either to *M. tuberculosis*, which synthesizes keto MA (kMA) or to *M. smegmatis*, which is devoid of kMA because the *hma* gene (Rv0642c, *mmaA4*) required for the production of oxygenated mycolic acid in pathogenic species, is not present in the non-pathogenic strain, *M. smegmatis*. FM were found only in granulomas prepared with *M. tuberculosis* or with recombinant strains of *M. smegmatis* over-expressing the *hma* gene product (Peyron et al., 2008).

Two recent studies confirmed that *in vitro*-grown human granulomas could be obtained by exposing PBMC to *M. tuberculosis* (Kapoor et al., 2013; Guirado et al., 2015). Using fluorescence microscopy approaches, Kapoor et al. confirmed that *M. tuberculosis* could accumulate lipids in the form of ILI within macrophages of the granulomatous structure. In addition, *M. tuberculosis* acquired other characteristics of dormant bacilli, such as loss of acid-fastness and tolerance to rifampicin (Kapoor et al., 2013). Treatment of such granulomas with monoclonal antibodies raised against tumor necrosis factor-alpha (TNFα), lead to resuscitation of bacilli and concurrent ILI consumption, suggestive of events resembling reactivation of TB. Guirado et al. identified the FM by staining the structures with Nile red (Guirado et al., 2015). They also showed that the amount of LB within FM was significantly higher when the PBMC used to construct the artificial human granulomas had been isolated from the blood of individuals with latent TB as opposed to that of healthy donors, thereby suggesting that differentiation of macrophages into FM is dependent on the host's immune status (Guirado et al., 2015).

A major advantage of the *in vitro*-grown human granuloma model, over *in vivo* animal models or *ex vivo* human biopsies, is the availability of live granuloma cells for analysis of their specific features and interactions with *M. tuberculosis* during persistence and reactivation. However, its complexity further restricts the panel of experimentations. For example, it is difficult to obtain preparations of either pure FM or of pure non-foamy macrophages and to undertake detailed kinetic studies of cellular events during transition from one to the other. In addition, this type of model adds limited clues with respect to lipid accumulation/consumption in mycobacteria, thus prompting several investigators to develop simpler, better defined and easier-to-handle foam cell models.

### Foam cell models

#### Cells cultured under conditions of hypoxia

In recent work, Daniel et al. (2011) analyzed the acquisition of dormancy characteristics by *M. tuberculosis* residing in cells cultured under conditions of hypoxia (Figure 2B). Their model was based on several observations among which: (i) caseous granulomas in the lungs of humans with untreated pulmonary TB contain lipid-loaded FM which harbor acid-fast bacilli (Hunter et al., 2007); (ii) such FM, which are found inside the hypoxic environment of TB granulomas, contain abundant stores of TAG and are thought to provide a lipid-rich environment for *M. tuberculosis* (Russell, 2007; Peyron et al., 2008); (iii) human macrophages cultured under conditions of hypoxia (1% O_2_) accumulate TAG in LB (Bostrom et al., 2006); and (iv) tuberculous granulomas in guinea pigs, rabbits and non-human primates are hypoxic (Via et al., 2008).

Two different types of macrophages were used in their study. In the first case, THP-1 monocytes were cultured in RPMI-1640 containing 10% fetal calf serum in a 5% CO_2_ incubator atmosphere at 37°C and differentiated into THP-M (THP-1 Macrophages), a frequently used macrophage-like cell line, obtained by stimulation with 100 nM phorbol 12-myristate 13-acetate (PMA) for 3 days. In the second case, the buffy coat obtained from Blood Centers after separation of other blood components was used for isolation of PBMC by density gradient centrifugation on Ficoll-Paque PLUS. PBMC were resuspended in RPMI-1640 and allowed to adhere onto plastic Petri dishes. Two hours later, non-adherent cells were removed and the adherent cells (mostly monocytes) were allowed to differentiate into macrophages over a period of 7 days under a 21% O_2_, 5% CO_2_ atmosphere in RPMI-1640 containing 10% human serum AB and 10 ng/ml granulocyte-macrophage colony stimulating factor (GM-CSF). Both types of macrophages were then infected with *M. tuberculosis* H37Rv at a MOI of either 0.1 or 5 bacilli per cell, and then incubated under hypoxia (1% O_2_, 5% CO_2_) for up to 5 days. Identical cells cultured in a normal environment (21% O_2_, 5% CO_2_) served as a control.

Under hypoxia conditions, both types of infected macrophages became foamy as shown by the accumulation of TAG-rich Oil-Red O-stained lipid droplets (Daniel et al., 2011). Inside such hypoxic FM, about half of the *M. tuberculosis* population developed tolerance to isoniazid, lost acid-fast staining and accumulated ILI. Moreover by disrupting the *Tgs1* gene, which is involved in TAG synthesis (Daniel et al., 2004; Sirakova et al., 2006; Viljoen et al., 2016), ILI formation and tolerance against antibiotics are reduced, suggesting that the lipid accumulation and drug tolerance are closely related (Baek et al., 2011). Subsequently, radioisotope and fluorescent fatty acid labeling of host TAG provided evidence for the utilization of the fatty acids from host TAG for lipid metabolism inside *M. tuberculosis*. Furthermore, reverse transcription PCR measurement revealed that several genes, known to be associated with dormancy and lipid metabolism, were upregulated in *M. tuberculosis* within the hypoxic lipid-loaded macrophages. Finally, *M. tuberculosis* was shown to replicate more slowly, thereby suggesting that the accumulation of TAG in mycobacterial ILI may be involved in mycobacterial growth. This latter result should, however, be interpreted with caution since at least 30% of the THP-M cells were no longer adherent by day 3 post-infection.

Cell growth under conditions of hypoxia represents an interesting approach for obtaining FM that closely resemble those formed under the hypoxic environment found in human tuberculous granulomas. However, to the extent that *in vitro* culturing of cells, and more especially of cell lines, such as THP-M, with or without hypoxia, may lead to cell death and the resulting release of fatty acids, the latter may serve as a source for TAG in both cellular LB and mycobacterial ILI rather than hypoxia itself. Likewise, *M. tuberculosis*, mostly in human macrophages, perturbs cellular lipid homeostasis, which results in the accumulation of LB in macrophages (Kim et al., 2010; Singh et al., 2012; Podinovskaia et al., 2013).

#### Cells exposed to fatty acids

##### Murine bone marrow-derived macrophages (BMDM) exposed to oleic acid

Several teams have shown that *M. tuberculosis* infection can lead to the retention of lipids and the maintenance of a foamy phenotype, notably when the host cell is a human monocyte-derived macrophages (HMDM) fed with human serum AB during culturing or within human *in vitro*-grown granulomas (Peyron et al., 2008).

The FM phenotype can also be induced by feeding BMDM with exogeneous fatty acids, such as oleic acid. In this experimental model (Lee et al., 2013; Podinovskaia et al., 2013) BMDM were incubated for 24 h in medium containing 400 μM oleate, complexed with BSA (Figure 2C). Cells were then infected with *M. tuberculosis* and the retention of the sequestered lipids was monitored for several days. The neutral lipids within LB were stained with the hydrophobic dye BODIPY 493/503 and observed by confocal microscopy. Comparable levels of fluorescent intensity were observed in both uninfected and infected macrophages after 24 h of exposure to oleate. Uninfected cells rapidly lost their LB content upon further culturing while those infected with *M. tuberculosis* retained the lipids within the LB, presumably due to reduced phagosomal lipolysis albeit many other factors may also be responsible for the decreased degradation/turnover of lipids in the infected cells.

Using the same model system, lipids in the form of ILI were also seen within *M. tuberculosis*, as reported previously (Peyron et al., 2008). To determine whether *M. tuberculosis* could have access to the host cell-derived lipids, cells were first infected with *M. tuberculosis* and incubated for 4 days to allow for the establishment of the infection. Cells were then exposed to oleate overnight and then pulsed with BODIPY FL-C16. This fluorescent dye was incorporated into both the host LB and the ILI within *M. tuberculosis*, thereby demonstrating that *M. tuberculosis* has access to lipids initially sequestered in host cell LB. However, how mycobacteria gain access to host lipids remained obscure.

##### Dictyostelium discoideum exposed to palmitic acid

The amoeba, *Dictyostelium discoideum*, widely used to characterize a variety of cellular processes, has been used recently to characterize the intracellular lipid metabolism and identify new proteins involved in lipid storage (Du et al., 2013).

**Figure 3:**
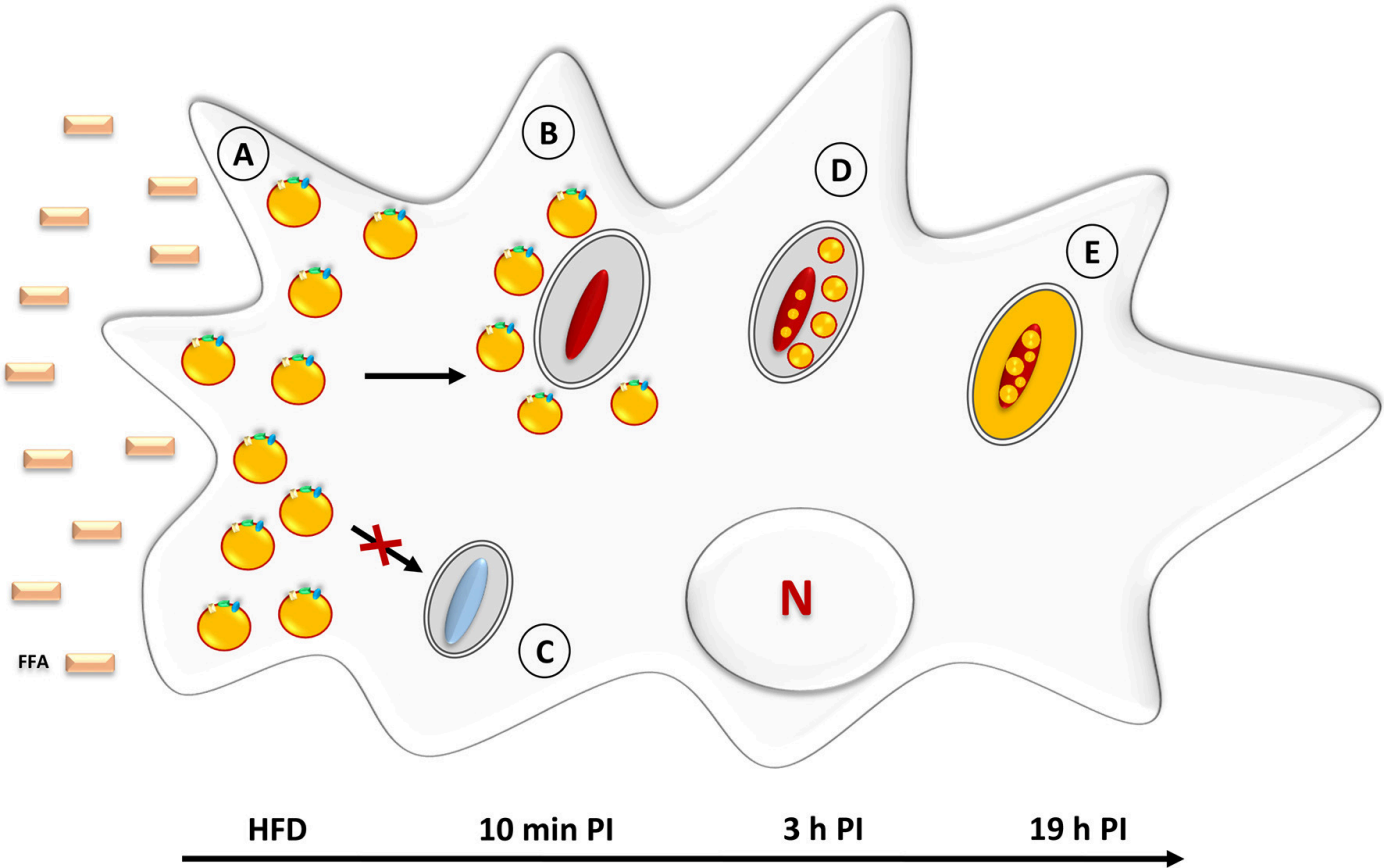
**Neutral lipid accumulation in *Mycobacterium marinum*-infected *D. discoideum***. **(A)** Prior to infection, amoebae are incubated with palmitic acid, corresponding to the High Fat Diet (HFD). This triggers the rapid formation of LB which leads to a foamy appearance. **(B)** Within 10 min post**-**infection with *M. marinum*, LB are gathered around the mycobacteria-containing vacuoles. **(C)** Infection with amikacin-killed mycobacteria fails to induce LB movement/relocation to the mycobacteria-containing vacuoles. **(D)** At 3 h post-infection, intact LB are found within the lumen of *M. marinum—*containing vacuoles. At this stage, a few ILI are already detectable inside the bacteria. **(E)** At later stages (19 h post-infection), neutral lipids are homogenously distributed inside the mycobacterial cytosol where they accumulate in the form of large ILI.

In order to induce the accumulation of lipid droplets in *D. discoideum*, the axenic growth medium was supplemented with 200 μM non-esterified palmitic acid C16 prepared from a 100 mM stock solution stored at −20°C. After 3 h of incubation in the presence of free-fatty acid (FFA), cells were re-incubated in fat-free medium. At selected time points thereafter, LB formation/degradation was monitored with Nile red, that serves to stain neutral lipids within LB, and further analyzed by thin layer chromatography (Du et al., 2013). Following exposure to palmitic acid for 3 h, the cells contained large amounts of LB and the TAG concentration had increased approximately 23-fold (Figure 3A; Du et al., 2013).

Based on these observations, Barisch et al. made use of this new foam cell model to characterize both the host and mycobacterial lipid metabolism upon *M. marinum* infection (Barisch et al., 2015). *D. discoideum* was first grown in axenic medium supplemented with 200 μM palmitic acid for 3 h. In some cases, BODIPY 558/568 C12 was added at a final concentration of 2 μM to label host TAG. After re-incubation in medium devoid of FFA, the cells were infected with *M. marinum*. Interactions between host LB and the *M. marinum*-containing vacuole were monitored by fluorescence microscopy and electron microscopy and time-lapse movies taken with a spinning disc confocal system. Strikingly, and within only 10 min post-infection, BODIPY-labeled LB, initially scattered in the host cell cytosol, started to cluster around *M. marinum*-containing vacuoles, and this process continued until significant depletion of LB in the host cell cytosol (Figure 3B; Barisch et al., 2015). When similar experiments were performed with amikacin-killed bacteria, LB were not recruited to the *M. marinum*-containing vacuoles (Figure 3C). That lipid droplets accumulate in the close vicinity of vacuoles containing live, but not dead, mycobacteria suggests an active recruitment of the LB. Mycobacterial factors secreted into the host cytosol may participate in this process. The authors reported that the amount of LB-associated BODIPY fluorescence in the cytoplasm of cells exposed to amikacin-killed *M. marinum* was not significantly different from that observed in uninfected cells (Barisch et al., 2015). Therefore, the absence of visible lipid droplets in the close vicinity of vacuoles containing dead bacilli could be related to the induction of a lower number of lipid droplets.

At 3 h post-infection, an accumulation of BODIPY-stained neutral lipids was observed in the lumen of the bacterium-containing vacuole (Figures 3D,E). EM analysis revealed also the presence of lipid and membrane-related structures within the *M. marinum*-containing vacuoles and, at later stages, ILI could be visualized inside the bacilli. These data suggest that the neutral lipids contained in host LB are delivered into the bacilli-containing vacuole from where they are transferred to mycobacteria where they accumulate in the form of ILI. In *D. discoideum* infected with *M. marinum*, it appears that the TAG-rich LB are engulfed by phagocytic vacuoles *via* a process that is reminiscent of autophagy (Peyron et al., 2008) rather than by direct fusion of LB with mycobacterium-containing phagosomes. The latter mechanism has been proven by EM approaches in another foam cell model (Caire-Brändli et al., 2014), described under section Cells Exposed to Lipoproteins.

Although the well-established model *D. discoideum* presents major assets for studying host-pathogen interplay, its main limitations reside in its growth conditions which are clearly different from those used to grow pathogenic mycobacteria. Whereas amoeba are grown at 22°C, *M. tuberculosis* has an optimal growth temperature of 37°C. Therefore, infections in *Dictyostelium* can only be performed with mycobacterial species growing at low temperatures, such as *M. marinum*, a species genetically related to *M. tuberculosis* and causing TB-like diseases in frogs and fish.

#### Cells exposed to lipoproteins

It has been suggested that the use of lipoprotein, rather than FFA, as an external source of lipids allows a lower lipid diffusion rate inside the host cell, a critical step for studying lipid metabolism of intracellular mycobacteria (Figure 2C; Dhouib et al., 2011; Caire-Brändli et al., 2014). Several investigators have, therefore, opted to expose mycobacterium-infected macrophages to lipoproteins, a choice guided by the fact that lipoproteins would provide lipids for the development of FM located close to the necrotic center of the granulomas (Peyron et al., 2008) where the caseum contains large amounts of cholesterol esters, cholesterol, TAG, and phospholipids from damaged/decaying cells (Kim et al., 2010). Lipids in lipoproteins that are internalized by scavenger receptor-mediated endocytosis undergo hydrolysis in lysosomes and provide the fatty acids for the subsequent biosynthesis of TAG in LB (Figure 2D).

**Figure 4:**
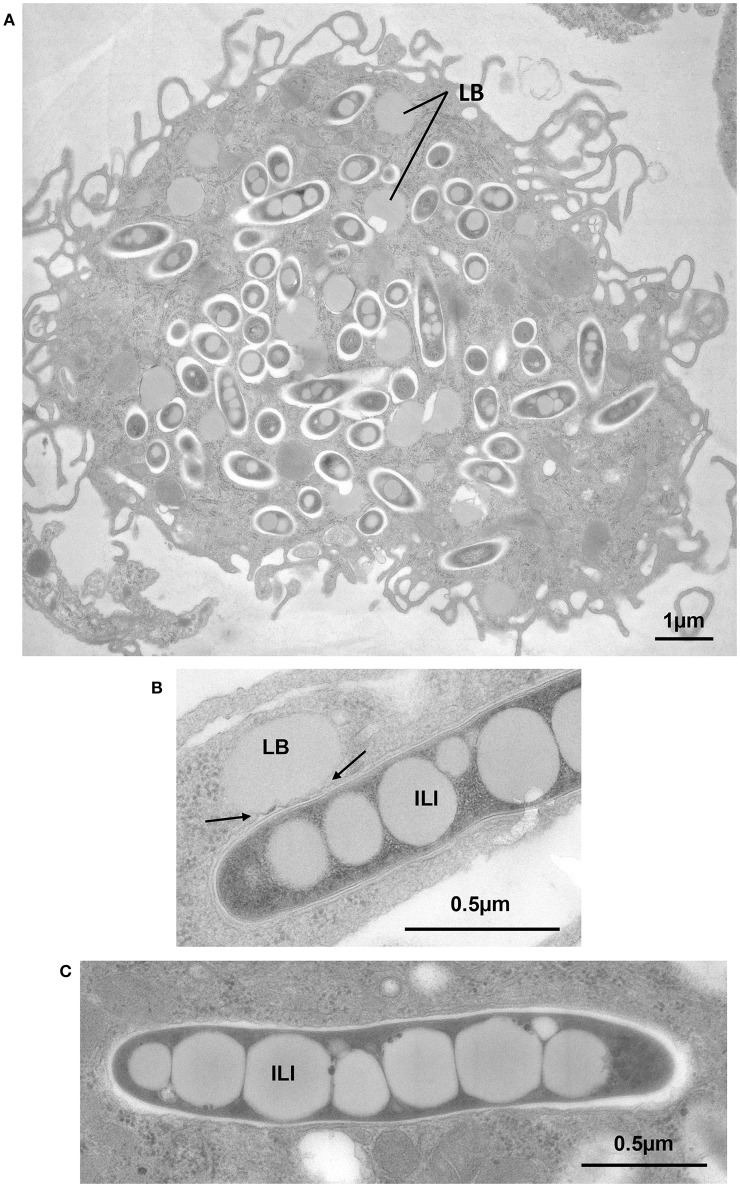
**Exposure of BMDM to VLDL induces foam cell formation, transfer of host TAG to mycobacterium-containing phagosomes by fusion of LB with phagosomes and accumulation of TAG in the form of ILI**. BMDM were infected with *M. avium or M. bovis* BCG. At day 6 post-infection., cells were exposed to VLDL for 24 h, fixed and processed for EM. **(A)** *M. avium*-infected cell displaying large amounts of LB, typical of FM. **(B)** BCG-containing phagosome in direct contact with an LB showing deformation of the phagosome membrane (arrows). **(C)** Intraphagosomal *M. avium* displaying several large ILI, 0.4 to 0.5 μm in width, and extending across the full width of the *M. avium* cytoplasm.

It is well known that when mycobacteria reside in phagosomes of foam cells, both the LB that give the cells their foamy appearance and the ILI accumulating in persistent bacilli, contain TAG as the major lipid component (Christensen et al., 1999; Tauchi-Sato et al., 2002; Wältermann and Steinbüchel, 2005). For this reason, a new experimental model of FM was developed (Caire-Brändli et al., 2014). In this model VLDL was preferred to low-density lipoprotein (LDL) or high-density lipoprotein (HDL) as an external source of lipid, because of its high TAG content (Mori et al., 2001). In contrast, LDL has a high sterol content and a low TAG content and HDL has a low content of both TAG and sterol. The experimental set-up consisted in first infecting BMDM with *M. avium* and, after active replication for 6 days, exposing the cells to VLDL as a lipid source (Caire-Brändli et al., 2014). Quantitative analysis of detailed EM observations combined with scoring of the number of CFU showed the following results: (i) macrophages became foamy (Figure 4A), and mycobacteria formed ILI (Figure 4C), for which host TAG, rather than cholesterol, was essential; (ii) host lipid transfer occurred *via* mycobacterium-induced fusion between LB and phagosomes (Figure 4B); (iii) mycobacteria displayed a thinned cell wall and became elongated but mycobacterial division was arrested; (iv) upon removal of VLDL, both the macrophage LB and the mycobacterial ILI declined within hours, and simultaneous resumption of mycobacterial division restored the number of bacteria to the same level as that found in untreated control macrophages (Caire-Brändli et al., 2014). From these data, the authors proposed that VLDL-driven FM constitute a well-defined cellular system in which to study changed metabolic states of intracellular mycobacteria that may relate to persistence and reactivation of TB.

The strength of this experimental system resides in its ability to generate defined conditions for triggering the formation or removal of LB, which induces ILI formation or consumption, respectively, and, in turn, leads to the reversible arrest of mycobacterial division without affecting bacterial growth. Furthermore, the use of EM approaches has allowed to studying major events at the subcellular level that cannot be observed by confocal microscopy, such as fusion of LB with mycobacterium-containing phagosomes or the alteration of the mycobacterial cell wall by thinning out of the electron-translucent outermost layer. The latter might result from an altered mycolic acid synthesis that might be responsible for loss of acid fastness and resistance to antibiotics (Bhatt et al., 2007; Vilchèze et al., 2014). Moreover, this approach allowed the authors to qualitatively demonstrate (i) incremental stages of ILI formation and, (ii) the presence or absence of septum formation in elongated cells. Importantly, these observations were sufficiently reliable and reproducible for quantitative analysis that allowed the authors to characterize the relationship between fluctuations of the ILI status and the arrest of mycobacterial division.

One may argue that under physiological conditions, neither monocytes nor lipoproteins will be found in the interstitium where a granuloma develops. Monocyte recruitment at such an inflammatory site may, however, be accompanied by an increased vascular permeability that would allow lipoproteins to leave the intravascular compartment, whereafter chemical modification enhances their uptake through scavenger receptors on macrophages.

In any case, this experimental model could be extremely beneficial for delineating the molecular mechanism responsible for the fusion of host LB with mycobacterium-containing phagosomes which leads to the translocation of host lipids to mycobacterium-containing phagosomes and for identifying the mycobacterial lipases involved in TAG accumulation and consumption during persistence and reactivation of TB.

### Adipocytes

About 15% of the reactivation cases of TB occur at extrapulmonary sites without an apparent pathology in the lungs (Wares et al., 2005; WHO, 2015). Many different organs and tissues are likely to host persistent *M. tuberculosis* during latency. Infection of non-immune cells with *M. tuberculosis* was, therefore, explored (Neyrolles et al., 2006; Randall et al., 2015). The adipose tissue, which is broadly distributed throughout the body, was likely to be a haven of choice for *M. tuberculosis* firstly because adipocytes display macrophage-like properties, such as the ability to phagocytose inert particles or living microorganisms, and, more especially because they could provide a natural lipid-rich environment for persistent bacilli (Cousin et al., 1999; Charrière et al., 2003; Neyrolles et al., 2006; Agarwal et al., 2014; Rastogi et al., 2016).

**Figure 5:**
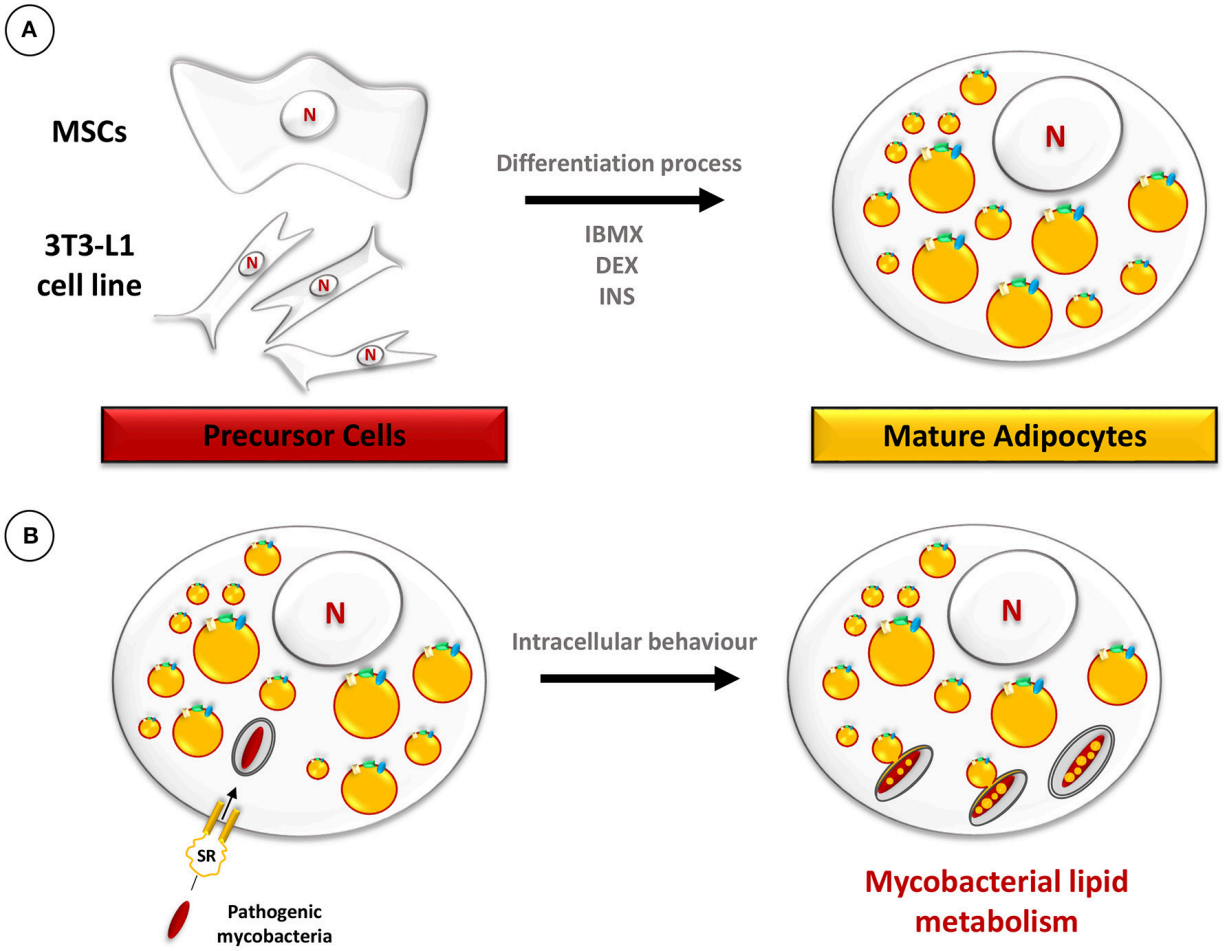
**Schematic representation of the adipocyte model. (A)** The differentiation procedure of precursor cells, such as mesenchymal stem cells (MSCs) or 3T3-L1, into mature adipocytes includes treatment with 3-isobutyl-1-methylxanthine (IBMX), dexamethasone (DEX), and insulin (INS). **(B)** Mature adipocytes, filled with LB, are infected with pathogenic mycobacteria which are phagocytosed after binding to scavenger receptors (Neyrolles et al., 2006). Ultimately, mycobacteria accumulate lipids in the form of ILI.

In this context, both the 3T3-L1 murine adipose cell line and human primary adipocytes were used as models to investigate interactions with *M. tuberculosis*. The immortalized 3T3-L1 murine cell line, derived from embryonic fibroblasts, has been widely used to obtain mature adipocytes by a chemically-induced differentiation process (Agarwal et al., 2014). The differentiation process of 3T3-L1 cells leads to the increased synthesis and huge accumulation of TAG in the form of LB (Figure 5A). Likewise, primary human adipocytes extracted from plastic surgery wastes can also be differentiated *in vitro* (Agarwal et al., 2014). During the infection process, *M. tuberculosis* is taken up by adipocytes of murine or human origin, after binding to scavenger receptors, and persists in a non-replicating state (Neyrolles et al., 2006). EM observations performed at 24 h post-infection, showed intracellular bacilli within membrane-bound vacuoles. A few bacilli were observed inside vacuoles whose electron translucent lumen was similar to that of LB. This observation added to the fact that the membrane of such vacuoles stained positively for perilipin, a specific surface marker of lipid droplets in adipocytes (Blanchette-Mackie et al., 1995), suggests that they most likely resulted from the fusion of adipocyte lipid droplets with the *M. tuberculosis*-containing vacuoles. Strikingly, bacilli within these vacuoles were heavily loaded with intracytosolic electron-translucent lipid inclusions, suggestive of a possible persistent phenotype (Figure 5B; Neyrolles et al., 2006). In agreement with these results, a more recent study proved that both *M. tuberculosis* H37Rv and H37Ra strains persist in a non-replicating state within adipocytes for up to 10 days post-infection (Kim et al., 2011). Interestingly, in this work observations of infected cells by confocal microscopy clearly showed intracellular bacilli in the close vicinity of LB, as demonstrated before with EM approaches (Neyrolles et al., 2006).

Finally, experimental infection of mature adipocytes directly isolated from the inguinal foot pads of mice or differentiated *ex vivo* from murine stromal vascular cells confirmed that *M. tuberculosis* infects natural murine adipocytes and pre-adipocytes (Agarwal et al., 2014). The fact that intra-adipocyte bacilli accumulate ILI starting from the third day post-infection, as assessed by a Nile red staining of neutral lipids (Agarwal et al., 2014), strengthens the validity of the 3T3-L1 murine cell model for studying lipid accumulation mechanisms leading to the persistence of *M. tuberculosis* outside the lungs. They also present the technical advantage of physiologically storing fat, thereby providing a natural lipid-rich environment, without additional induction steps to provide a foamy phenotype. However, once differentiated into mature adipocytes, filled with LB, the cells cannot revert back to a non-foamy phenotype. Therefore, this model is suitable for studying the accumulation of mycobacterial lipids but the fact that the cellular lipid-rich environment is non-reversible make this system inappropriate for investigating lipid consumption within mycobacteria.

## Concluding remarks and future prospects

The different models presented in the present review have allowed to obtain relevant information on lipid metabolism in the context of *M. tuberculosis* persistence and reactivation. The use of animal models and *ex vivo* experimental systems have led to breakthrough information regarding cellular and molecular aspects related to (i) the acquisition of a foam cell phenotype where cells can be induced to store fat within LB under specific stimuli; (ii) the transfer of host LB contents to the lumen of the mycobacterium-containing phagosome following fusion events between LB and phagosomes, and (iii) the accumulation of neutral lipids in the form of ILI within mycobacteria.

Indeed, this large number of well-established host-pathogens systems, has allowed, recently the identification of a wide range of macrophages genes which are directly involved into pro-inflammatory response, energetic metabolism, such as glucose and lipid anabolism/catabolism leading to a foamy phenotype (Singh et al., 2015; Bhagyaraj et al., 2016; Holla et al., 2016).

In addition to those host factors, several mycobacterial proteins are likely to be involved in these various processes and identified (Deb et al., 2006; Kim et al., 2010; Low et al., 2010; Daniel et al., 2016).

As pointed out by Höner Zu Bentrup and Russell (2001), many genes of the *M. tuberculosis* genome encode enzymes involved in lipid metabolism (Cole et al., 1998). Among these, several might play important roles in the above-mentioned processes, particularly in the fusion events between LB and mycobacterium-containing phagosomes and the release of host lipids. As proposed by Caire-Brändli et al. (2014), these events can be viewed as the reverse steps involved in the transfer of lipids from the ER into the cytosol in the form of LB. The phospholipid monolayer leaflet covering the entire LB could merge with the outer leaflet of the phagosome membrane bilayer. This would leave the lipid contents between the two leaflets of the phagosomal membrane bilayer, where they remain separated from the mycobacterial surface by the inner leaflet. Because this remaining phospholipid barrier would present a different molecular arrangement than the otherwise intact phagosome membrane bilayer, it might be more vulnerable to the attack by mycobacterial lipolytic enzymes. Several mycobacteria, like *M. avium, M. tuberculosis*, and *M. bovis* BCG, seem to use essentially the neutral lipids contained within LB as a source of carbon during persistence, by secreting their own lipolytic enzymes and by activating host lipolytic enzymes (Daniel et al., 2011; Singh et al., 2012). In this context, *M. tuberculosis* genes encoding lipid/ester hydrolases, with a wide range of hydrolytic activities (Dedieu et al., 2013), deserve special attention. These enzymes display specific properties, such as the ability to be secreted and the capacity to hydrolyze phospholipid membrane layers. Cytotoxic effects have also been associated with some of these enzymes (Bakala N'goma et al., 2010; Schué et al., 2010). We propose that these enzymes attack the inner leaflet of the phagosome membrane bilayer. As a consequence, the LB content is released inside the phagosomal lumen, in the immediate vicinity of the mycobacterial surface. EM analyses also revealed that TAG from LB are rapidly re-used to form ILI, indicating that these molecules are first hydrolyzed to produce FFA and then imported inside the bacterial cytosol (Caire-Brändli et al., 2014). This implies the contribution of mycobacterial lipases exposed at the cell wall surface. The *HSL*-homologe LipY (Rv3097c), which hydrolyses long fatty acyl chains, is secreted by a type VII secretion system (ESX-5). LipY remains one of the most studied lipase of *M. tuberculosis* (Deb et al., 2006; Mishra et al., 2008; Delorme et al., 2012). Deletion of the *lipY* gene is accompanied by a significant decrease in the amount of ILI, indicating that LipY participates in TAG hydrolysis (Deb et al., 2006), although additional lipolytic activities are likely to be involved (Côtes et al., 2007; Dhouib et al., 2010; Schué et al., 2010). Recent transcriptomic studies in adipocytes infected with *M. tuberculosis* also favored the potential involvement of various *M. tuberculosis* lipases, cutinases, and esterases in lipid accumulation/consumption processes (Rastogi et al., 2016).

How the major lipolysis products derived from host TAG (DAG, MAG, and FFA) are formed and how they gain access to the mycobacterial cytosol is not fully understood. Over the past few years, several membrane proteins have been identified as potential actors of nutrient uptake and/or lipid mobilization in mycobacteria (Daniel et al., 2014; Ates et al., 2015; Martinot et al., 2016). Interestingly, a specific class of fatty acid transporter proteins (FATP), conserved in mycobacteria and humans, has been identified as a potential actor in the mobilization and activation of FFA (Hirsch et al., 1998; Daniel et al., 2014) leading to TAG synthesis by bacterial di and triacylglycerol synthases (Daniel et al., 2004; Sirakova et al., 2006; Viljoen et al., 2016).

We anticipate that the use of these various and complementary models and approaches described in the present review will undoubtedly facilitate the discovery of new cellular events and/or mycobacterial factors that will help to further delineate the intricate mechanisms involved in mycobacterial lipid acquisition and assimilation during the intracellular lifestyle of *M. tuberculosis*.

## Author contributions

PS participated in the drafting of the section on lipid accumulation in macrophages, FB on adipocytes and NS on Dictyostelium parts. The introduction section was written by PS and FB. The figures were drawn by PS and electron microscopy pictures were provided by CD and IP. MD was helpful for correcting the manuscript. LK, CD, and SC completed and revised the entire manuscript. All of this work was proposed and supervised by SC.

### Conflict of interest statement

The authors declare that the research was conducted in the absence of any commercial or financial relationships that could be construed as a potential conflict of interest.

## References

[B1] Agarwal P. Khan S. R. Verma S. C. Beg M. Singh K. Mitra K.. (2014). *Mycobacterium* tuberculosis persistence in various adipose depots of infected mice and the effect of anti-tubercular therapy. Microbes Infect. 16, 571–580. 10.1016/j.micinf.2014.04.006 24819214

[B2] Alvarez H. M. (2016). Triacylglycerol and wax ester-accumulating machinery in prokaryotes. Biochimie 120, 28–39. 10.1016/j.biochi.2015.08.016 26343555

[B3] Ates L. S. Ummels R. Commandeur S. Van De Weerd R. Sparrius M. Weerdenburg E.. (2015). Essential role of the ESX-5 secretion system in outer membrane permeability of pathogenic *Mycobacteria*. PLoS Genet. 11:e1005190. 10.1371/journal.pgen.1005190 25938982 PMC4418733

[B4] Baek S. H. Li A. H. Sassetti C. M. (2011). Metabolic regulation of mycobacterial growth and antibiotic sensitivity. PLoS Biol. 9:e1001065. 10.1371/journal.pbio.1001065 21629732 PMC3101192

[B5] Bakala N'goma J. C. Schué M. Carriere F. Geerlof A. Canaan S. (2010). Evidence for the cytotoxic effects of *Mycobacterium tuberculosis* phospholipase C towards macrophages. Biochim. Biophys. Acta 1801, 1305–1313. 10.1016/j.bbalip.2010.08.007 20736081

[B6] Barisch C. Paschke P. Hagedorn M. Maniak M. Soldati T. (2015). Lipid droplet dynamics at early stages of *Mycobacterium marinum* infection in Dictyostelium. Cell. Microbiol. 17, 1332–1349. 10.1111/cmi.12437 25772333

[B7] Beatty W. L. Rhoades E. R. Ullrich H. J. Chatterjee D. Heuser J. E. Russell D. G. (2000). Trafficking and release of mycobacterial lipids from infected macrophages. Traffic 1, 235–247. 10.1034/j.1600-0854.2000.010306.x 11208107

[B8] Bhagyaraj E. Nanduri R. Saini A. Dkhar H. K. Ahuja N. Chandra V.. (2016). Human Xenobiotic Nuclear Receptor PXR augments *Mycobacterium tuberculosis* survival. J. Immunol. 197, 244–255. 10.4049/jimmunol.1600203 27233963

[B9] Bhatt A. Fujiwara N. Bhatt K. Gurcha S. S. Kremer L. Chen B.. (2007). Deletion of kasB in *Mycobacterium tuberculosis* causes loss of acid-fastness and subclinical latent tuberculosis in immunocompetent mice. Proc. Natl. Acad. Sci. U.S.A. 104, 5157–5162. 10.1073/pnas.0608654104 17360388 PMC1829279

[B10] Blanchette-Mackie E. J. Dwyer N. K. Barber T. Coxey R. A. Takeda T. Rondinone C. M.. (1995). Perilipin is located on the surface layer of intracellular lipid droplets in adipocytes. J. Lipid Res. 36, 1211–1226. 7665999

[B11] Bostrom P. Magnusson B. Svensson P. A. Wiklund O. Borén J. Carlsson L. M.. (2006). Hypoxia converts human macrophages into triglyceride-loaded foam cells. Arterioscler. Thromb. Vasc. Biol. 26, 1871–1876. 10.1161/01.ATV.0000229665.78997.0b 16741148

[B12] Brasaemle D. L. Dolios G. Shapiro L. Wang R. (2004). Proteomic analysis of proteins associated with lipid droplets of basal and lipolytically stimulated 3T3-L1 adipocytes. J. Biol. Chem. 279, 46835–46842. 10.1074/jbc.M409340200 15337753

[B13] Cáceres N. Tapia G. Ojanguren I. Altare F. Gil O. Pinto S.. (2009). Evolution of foamy macrophages in the pulmonary granulomas of experimental tuberculosis models. Tuberculosis (Edinb). 89, 175–182. 10.1016/j.tube.2008.11.001 19110471

[B14] Caire-Brändli I. Papadopoulos A. Malaga W. Marais D. Canaan S. Thilo L.. (2014). Reversible lipid accumulation and associated division arrest of *Mycobacterium* avium in lipoprotein-induced foamy macrophages may resemble key events during latency and reactivation of tuberculosis. Infect. Immun. 82, 476–490. 10.1128/IAI.01196-13 24478064 PMC3911402

[B15] Cardona P. J. Llatjós R. Gordillo S. Díaz J. Ojanguren I. Ariza A.. (2000). Evolution of granulomas in lungs of mice infected aerogenically with *Mycobacterium tuberculosis*. Scand. J. Immunol. 52, 156–163. 10.1046/j.1365-3083.2000.00763.x 10931383

[B16] Cardoso M. S. Silva T. M. Resende M. Appelberg R. Borges M. (2015). Lack of the transcription factor hypoxia-inducible factor 1alpha (HIF-1alpha) in macrophages accelerates the necrosis of *Mycobacterium* avium-induced granulomas. Infect. Immun. 83, 3534–3544. 10.1128/IAI.00144-15 26099585 PMC4534669

[B17] Charrière G. Cousin B. Arnaud E. Andrè M. Bacou F. Penicaud L.. (2003). Preadipocyte conversion to macrophage. Evidence of plasticity. J. Biol. Chem. 278, 9850–9855. 10.1074/jbc.M210811200 12519759

[B18] Christensen H. Garton N. J. Horobin R. W. Minnikin D. E. Barer M. R. (1999). Lipid domains of mycobacteria studied with fluorescent molecular probes. Mol. Microbiol. 31, 1561–1572. 10.1046/j.1365-2958.1999.01304.x 10200973

[B19] Cole S. T. Brosch R. Parkhill J. Garnier T. Churcher C. Harris D.. (1998). Deciphering the biology of *Mycobacterium tuberculosis* from the complete genome sequence. Nature 393, 537–544. 10.1038/31159 9634230

[B20] Côtes K. Dhouib R. Douchet I. Chahinian H. De Caro A. Carrière F.. (2007). Characterization of an exported monoglyceride lipase from *Mycobacterium tuberculosis* possibly involved in the metabolism of host cell membrane lipids. Biochem. J. 408, 417–427. 10.1042/BJ20070745 17784850 PMC2267359

[B21] Cousin B. Munoz O. Andre M. Fontanilles A. M. Dani C. Cousin J. L.. (1999). A role for preadipocytes as macrophage-like cells. FASEB J. 13, 305–312. 9973318 10.1096/fasebj.13.2.305

[B22] Daniel J. Deb C. Dubey V. S. Sirakova T. D. Abomoelak B. Morbidoni H. R.. (2004). Induction of a novel class of diacylglycerol acyltransferases and triacylglycerol accumulation in *Mycobacterium tuberculosis* as it goes into a dormancy-like state in culture. J. Bacteriol. 186, 5017–5030. 10.1128/JB.186.15.5017-5030.2004 15262939 PMC451596

[B23] Daniel J. Kapoor N. Sirakova T. Sinha R. Kolattukudy P. (2016). The perilipin-like PPE15 protein in *Mycobacterium tuberculosis* is required for triacylglycerol accumulation under dormancy-inducing conditions. Mol. Microbiol. 101, 784–794. 10.1111/mmi.13422 27325376 PMC5019126

[B24] Daniel J. Maamar H. Deb C. Sirakova T. D. Kolattukudy P. E. (2011). *Mycobacterium tuberculosis* uses host triacylglycerol to accumulate lipid droplets and acquires a dormancy-like phenotype in lipid-loaded macrophages. PLoS Pathog. 7:e1002093. 10.1371/journal.ppat.1002093 21731490 PMC3121879

[B25] Daniel J. Sirakova T. Kolattukudy P. (2014). An acyl-CoA synthetase in *Mycobacterium tuberculosis* involved in triacylglycerol accumulation during dormancy. PLoS ONE 9:e114877. 10.1371/journal.pone.0114877 25490545 PMC4260918

[B26] Datta M. Via L. E. Chen W. Baish J. W. Xu L. Barry C. E. III. (2016). Mathematical model of oxygen transport in tuberculosis granulomas. Ann. Biomed. Eng. 44, 863–872. 10.1007/s10439-015-1415-3 26253038 PMC4795989

[B27] Deb C. Daniel J. Sirakova T. D. Abomoelak B. Dubey V. S. Kolattukudy P. E. (2006). A novel lipase belonging to the hormone-sensitive lipase family induced under starvation to utilize stored triacylglycerol in *Mycobacterium tuberculosis*. J. Biol. Chem. 281, 3866–3875. 10.1074/jbc.M505556200 16354661 PMC1523426

[B28] Dedieu L. Serveau-Avesque C. Kremer L. Canaan S. (2013). Mycobacterial lipolytic enzymes: a gold mine for tuberculosis research. Biochimie 95, 66–73. 10.1016/j.biochi.2012.07.008 22819994

[B29] Delorme V. Diomandè S. V. Dedieu L. Cavalier J. F. Carrière F. Kremer L.. (2012). MmPPOX Inhibits *Mycobacterium tuberculosis* lipolytic enzymes belonging to the hormone-sensitive lipase family and alters mycobacterial growth. PLoS ONE 7:e46493. 10.1371/journal.pone.0046493 23029536 PMC3460867

[B30] Dhouib R. Ducret A. Hubert P. Carrière F. Dukan S. Canaan S. (2011). Watching intracellular lipolysis in mycobacteria using time lapse fluorescence microscopy. Biochim. Biophys. Acta 1811, 234–241. 10.1016/j.bbalip.2011.01.001 21238605

[B31] Dhouib R. Laval F. Carrière F. Daffe M. Canaan S. (2010). A monoacylglycerol lipase from *Mycobacterium smegmatis* Involved in bacterial cell interaction. J. Bacteriol. 192, 4776–4785. 10.1128/JB.00261-10 20601476 PMC2937407

[B32] Dkhar H. K. Nanduri R. Mahajan S. Dave S. Saini A. Somavarapu A. K.. (2014). *Mycobacterium tuberculosis* keto-mycolic acid and macrophage nuclear receptor TR4 modulate foamy biogenesis in granulomas: a case of a heterologous and noncanonical ligand-receptor pair. J. Immunol. 193, 295–305. 10.4049/jimmunol.1400092 24907344

[B33] Driver E. R. Ryan G. J. Hoff D. R. Irwin S. M. Basaraba R. J. Kramnik I.. (2012). Evaluation of a mouse model of necrotic granuloma formation using C3HeB/FeJ mice for testing of drugs against *Mycobacterium tuberculosis*. Antimicrobial Agents Chemother. 56, 3181–3195. 10.1128/AAC.00217-12 22470120 PMC3370740

[B34] Du X. Barisch C. Paschke P. Herrfurth C. Bertinetti O. Pawolleck N.. (2013). Dictyostelium lipid droplets host novel proteins. Eukaryot. Cell 12, 1517–1529. 10.1128/EC.00182-13 24036346 PMC3837934

[B35] Feng Y. Dorhoi A. Mollenkopf H. J. Yin H. Dong Z. Mao L.. (2014). Platelets direct monocyte differentiation into epithelioid-like multinucleated giant foam cells with suppressive capacity upon mycobacterial stimulation. J. Infect. Dis. 210, 1700–1710. 10.1093/infdis/jiu355 24987031 PMC4224136

[B36] Florey H. (1958). Tuberculosis, in General Pathology Based on Lectures Delivered at the Sir William Dunn School of Pathology, ed Saunders W. B. (Oxford: University of Oxford), 829–870.

[B37] Galagan J. E. (2014). Genomic insights into tuberculosis. Nat. Rev. Genet. 15, 307–320. 10.1038/nrg3664 24662221

[B38] Garton N. J. Christensen H. Minnikin D. E. Adegbola R. A. Barer M. R. (2002). Intracellular lipophilic inclusions of mycobacteria *in vitro* and in sputum. Microbiology 148, 2951–2958. 10.1099/00221287-148-10-2951 12368428

[B39] Geisel R. E. Sakamoto K. Russell D. G. Rhoades E. R. (2005). *In vivo* activity of released cell wall lipids of *Mycobacterium bovis* bacillus Calmette-Guerin is due principally to trehalose mycolates. J. Immunol. 174, 5007–5015. 10.4049/jimmunol.174.8.5007 15814731

[B40] Gideon H. P. Flynn J. L. (2011). Latent tuberculosis: what the host “sees”? Immunol. Res. 50, 202–212. 10.1007/s12026-011-8229-7 21717066 PMC3788603

[B41] Glatz J. F. Luiken J. J. Bonen A. (2010). Membrane fatty acid transporters as regulators of lipid metabolism: implications for metabolic disease. Physiol. Rev. 90, 367–417. 10.1152/physrev.00003.2009 20086080

[B42] Guirado E. Mbawuike U. Keiser T. L. Arcos J. Azad A. K. Wang S. H.. (2015). Characterization of host and microbial determinants in individuals with latent tuberculosis infection using a human granuloma model. MBio 6, e02537–e02514. 10.1128/mBio.02537-14 25691598 PMC4337582

[B43] Guirado E. Schlesinger L. S. (2013). Modeling the *Mycobacterium tuberculosis* granuloma - the critical battlefield in host immunity and disease. Front. Immunol. 4:98. 10.3389/fimmu.2013.00098 23626591 PMC3631743

[B44] Harris C. A. Haas J. T. Streeper R. S. Stone S. J. Kumari M. Yang K.. (2011). DGAT enzymes are required for triacylglycerol synthesis and lipid droplets in adipocytes. J. Lipid Res. 52, 657–667. 10.1194/jlr.M013003 21317108 PMC3284159

[B45] Hingley-Wilson S. M. Sambandamurthy V. K. Jacobs W. R. Jr. (2003). Survival perspectives from the world's most successful pathogen, *Mycobacterium tuberculosis*. Nat. Immunol. 4, 949–955. 10.1038/ni981 14515128

[B46] Hirsch D. Stahl A. Lodish H. F. (1998). A family of fatty acid transporters conserved from *mycobacterium* to man. Proc. Natl. Acad. Sci. U.S.A. 95, 8625–8629. 10.1073/pnas.95.15.8625 9671728 PMC21126

[B47] Holla S. Prakhar P. Singh V. Karnam A. Mukherjee T. Mahadik K.. (2016). MUSASHI-mediated expression of JMJD3, a H3K27me3 demethylase, is involved in foamy macrophage generation during mycobacterial infection. PLoS Pathog. 12:e1005814. 10.1371/journal.ppat.1005814 27532872 PMC4988650

[B48] Höner Zu Bentrup K. Russell D. G. (2001). Mycobacterial persistence: adaptation to a changing environment. Trends Microbiol. 9, 597–605. 10.1016/S0966-842X(01)02238-7 11728873

[B49] Hunter R. L. Jagannath C. Actor J. K. (2007). Pathology of postprimary tuberculosis in humans and mice: contradiction of long-held beliefs. Tuberculosis (Edinb). 87, 267–278. 10.1016/j.tube.2006.11.003 17369095

[B50] Irwin S. M. Driver E. Lyon E. Schrupp C. Ryan G. Gonzalez-Juarrero M.. (2015). Presence of multiple lesion types with vastly different microenvironments in C3HeB/FeJ mice following aerosol infection with *Mycobacterium tuberculosis*. Dis. Model. Mech. 8, 591–602. 10.1242/dmm.019570 26035867 PMC4457037

[B51] Jain M. Petzold C. J. Schelle M. W. Leavell M. D. Mougous J. D. Bertozzi C. R.. (2007). Lipidomics reveals control of *Mycobacterium tuberculosis* virulence lipids via metabolic coupling. Proc. Natl. Acad. Sci. U.S.A. 104, 5133–5138. 10.1073/pnas.0610634104 17360366 PMC1829275

[B52] Kapoor N. Pawar S. Sirakova T. D. Deb C. Warren W. L. Kolattukudy P. E. (2013). Human granuloma *in vitro* model, for TB dormancy and resuscitation. PLoS ONE 8:e53657. 10.1371/journal.pone.0053657 23308269 PMC3538642

[B53] Karakousis P. C. Yoshimatsu T. Lamichhane G. Woolwine S. C. Nuermberger E. L. Grosset J.. (2004). Dormancy phenotype displayed by extracellular *Mycobacterium tuberculosis* within artificial granulomas in mice. J. Exp. Med. 200, 647–657. 10.1084/jem.20040646 15353557 PMC2212740

[B54] Kassan A. Herms A. Fernández-Vidal A. Bosch M. Schieber N. L. Reddy B. J.. (2013). Acyl-CoA synthetase 3 promotes lipid droplet biogenesis in ER microdomains. J. Cell Biol. 203, 985–1001. 10.1083/jcb.201305142 24368806 PMC3871434

[B55] Kaufmann S. H. (2001). How can immunology contribute to the control of tuberculosis? Nat. Rev. Immunol. 1, 20–30. 10.1038/35095558 11905811

[B56] Keller C. Lauber J. Blumenthal A. Buer J. Ehlers S. (2004). Resistance and susceptibility to tuberculosis analysed at the transcriptome level: lessons from mouse macrophages. Tuberculosis 84, 144–158. 10.1016/j.tube.2003.12.003 15207484

[B57] Kim J. S. Ryu M. J. Byun E. H. Kim W. S. Whang J. Min K. N.. (2011). Differential immune response of adipocytes to virulent and attenuated *Mycobacterium tuberculosis*. Microbes Infect. 13, 1242–1251. 10.1016/j.micinf.2011.07.002 21813088

[B58] Kim M. J. Wainwright H. C. Locketz M. Bekker L. G. Walther G. B. Dittrich C.. (2010). Caseation of human tuberculosis granulomas correlates with elevated host lipid metabolism. EMBO Mol. Med. 2, 258–274. 10.1002/emmm.201000079 20597103 PMC2913288

[B59] Korf J. Stoltz A. Verschoor J. De Baetselier P. Grooten J. (2005). The *Mycobacterium tuberculosis* cell wall component mycolic acid elicits pathogen-associated host innate immune responses. Eur. J. Immunol. 35, 890–900. 10.1002/eji.200425332 15724242

[B60] Kramnik I. Demant P. Bloom B. B. (1998). Susceptibility to tuberculosis as a complex genetic trait: analysis using recombinant congenic strains of mice. Novartis Found. Symp. 217, 120–131; discussion 132–127. 9949805 10.1002/0470846526.ch9

[B61] Kramnik I. Dietrich W. F. Demant P. Bloom B. R. (2000). Genetic control of resistance to experimental infection with virulent *Mycobacterium tuberculosis*. Proc. Natl. Acad. Sci. U.S.A. 97, 8560–8565. 10.1073/pnas.150227197 10890913 PMC26987

[B62] Kumar A. Farhana A. Guidry L. Saini V. Hondalus M. Steyn A. J. (2011). Redox homeostasis in mycobacteria: the key to tuberculosis control? Expert Rev. Mol. Med. 13, e39. 10.1017/S1462399411002079 22172201 PMC3241215

[B63] Lee W. Vanderven B. C. Fahey R. J. Russell D. G. (2013). Intracellular *Mycobacterium tuberculosis* exploits host-derived fatty acids to limit metabolic stress. J. Biol. Chem. 288, 6788–6800. 10.1074/jbc.M112.445056 23306194 PMC3591590

[B64] Listenberger L. L. Han X. Lewis S. E. Cases S. Farese R. V. Jr. Ory D. S.. (2003). Triglyceride accumulation protects against fatty acid-induced lipotoxicity. Proc. Natl. Acad. Sci. U.S.A. 100, 3077–3082. 10.1073/pnas.0630588100 12629214 PMC152249

[B65] Low K. L. Rao P. S. Shui G. Bendt A. K. Pethe K. Dick T. (2009). Triacylglycerol utilization is required for regrowth of *in vitro* hypoxic non-replicating *Mycobacterium bovis* bacillus Calmette-Guerin. J. Bacteriol. 191, 5037–5043. 10.1128/JB.00530-09 19525349 PMC2725574

[B66] Low K. L. Shui G. Natter K. Yeo W. K. Kohlwein S. D. Dick T.. (2010). Lipid droplet-associated proteins are involved in the biosynthesis and hydrolysis of triacylglycerol in *Mycobacterium bovis* bacillus Calmette-Guerin. J. Biol. Chem. 285, 21662–21670. 10.1074/jbc.M110.135731 20452973 PMC2898439

[B67] Mahajan S. Dkhar H. K. Chandra V. Dave S. Nanduri R. Janmeja A. K.. (2012). *Mycobacterium tuberculosis* modulates macrophage lipid-sensing nuclear receptors PPARgamma and TR4 for survival. J. Immunol. 188, 5593–5603. 10.4049/jimmunol.1103038 22544925

[B68] Martin S. Parton R. G. (2005). Caveolin, cholesterol, and lipid bodies. Semin. Cell Dev. Biol. 16, 163–174. 10.1016/j.semcdb.2005.01.007 15797827

[B69] Martinot A. J. Farrow M. Bai L. Layre E. Cheng T. Y. Tsai J. H.. (2016). Mycobacterial metabolic syndrome: LprG and Rv1410 regulate triacylglyceride levels, growth rate and virulence in *Mycobacterium tuberculosis*. PLoS Pathog. 12:e1005351. 10.1371/journal.ppat.1005351 26751071 PMC4709180

[B70] Mattos K. A. D'avila H. Rodrigues L. S. Oliveira V. G. Sarno E. N. Atella G. C.. (2010). Lipid droplet formation in leprosy: toll-like receptor-regulated organelles involved in eicosanoid formation and *Mycobacterium leprae* pathogenesis. J. Leukoc. Biol. 87, 371–384. 10.1189/jlb.0609433 19952355

[B71] Mattos K. A. Lara F. A. Oliveira V. G. Rodrigues L. S. D'avila H. Melo R. C.. (2011). Modulation of lipid droplets by *Mycobacterium leprae* in Schwann cells: a putative mechanism for host lipid acquisition and bacterial survival in phagosomes. Cell. Microbiol. 13, 259–273. 10.1111/j.1462-5822.2010.01533.x 20955239

[B72] Mckinney J. D. Gomez J. E. (2003). Life on the inside for *Mycobacterium tuberculosis*. Nat. Med. 9, 1356–1357. 10.1038/nm1103-1356 14595429

[B73] Mckinney J. D. Höner Zu Bentrup K. Muñoz-Elías E. J. Miczak A. Chen B. Chan W. T.. (2000). Persistence of *Mycobacterium tuberculosis* in macrophages and mice requires the glyoxylate shunt enzyme isocitrate lyase. Nature 406, 735–738. 10.1038/35021074 10963599

[B74] Mishra K. C. De Chastellier C. Narayana Y. Bifani P. Brown A. K. Besra G. S.. (2008). Functional role of the PE domain and immunogenicity of the *Mycobacterium tuberculosis* triacylglycerol hydrolase LipY. Infect. Immun. 76, 127–140. 10.1128/IAI.00410-07 17938218 PMC2223678

[B75] Mori M. Itabe H. Higashi Y. Fujimoto Y. Shiomi M. Yoshizumi M.. (2001). Foam cell formation containing lipid droplets enriched with free cholesterol by hyperlipidemic serum. J. Lipid Res. 42, 1771–1781. 11714846

[B76] Murphy D. J. (2001). The biogenesis and functions of lipid bodies in animals, plants and microorganisms. Prog. Lipid Res. 40, 325–438. 10.1016/S0163-7827(01)00013-3 11470496

[B77] Murphy D. J. Vance J. (1999). Mechanisms of lipid-body formation. Trends Biochem. Sci. 24, 109–115. 10.1016/S0968-0004(98)01349-8 10203758

[B78] Neyrolles O. Hernández-Pando R. Pietri-Rouxel F. Fornes P. Tailleux L. Payan J. A.. (2006). Is adipose tissue a place for *Mycobacterium tuberculosis* persistence? PLoS ONE 1:e43. 10.1371/journal.pone.0000043 17183672 PMC1762355

[B79] Ohsaki Y. Cheng J. Suzuki M. Shinohara Y. Fujita A. Fujimoto T. (2009). Biogenesis of cytoplasmic lipid droplets: from the lipid ester globule in the membrane to the visible structure. Biochim. Biophys. Acta 1791, 399–407. 10.1016/j.bbalip.2008.10.002 18996222

[B80] Ordway D. Harton M. Henao-Tamayo M. Montoya R. Orme I. M. Gonzalez-Juarrero M. (2006). Enhanced macrophage activity in granulomatous lesions of immune mice challenged with *Mycobacterium tuberculosis*. J. Immunol. 176, 4931–4939. 10.4049/jimmunol.176.8.4931 16585589

[B81] Ordway D. Henao-Tamayo M. Orme I. M. Gonzalez-Juarrero M. (2005). Foamy macrophages within lung granulomas of mice infected with *Mycobacterium tuberculosis* express molecules characteristic of dendritic cells and antiapoptotic markers of the TNF receptor-associated factor family. J. Immunol. 175, 3873–3881. 10.4049/jimmunol.175.6.3873 16148133

[B82] Pagel W. (1925). Fat and lipoid content to tuberculous tissue: Histochemical investigation. Virschows Arch. Path. Anat. 256, 629–640. 10.1007/BF01891650

[B83] Peyron P. Vaubourgeix J. Poquet Y. Levillain F. Botanch C. Bardou F.. (2008). Foamy macrophages from tuberculous patients' granulomas constitute a nutrient-rich reservoir for *M. tuberculosis* persistence. PLoS Pathog. 4:e1000204. 10.1371/journal.ppat.1000204 19002241 PMC2575403

[B84] Pichugin A. V. Yan B. S. Sloutsky A. Kobzik L. Kramnik I. (2009). Dominant role of the sst1 locus in pathogenesis of necrotizing lung granulomas during chronic tuberculosis infection and reactivation in genetically resistant hosts. Am. J. Pathol. 174, 2190–2201. 10.2353/ajpath.2009.081075 19443700 PMC2684184

[B85] Podinovskaia M. Lee W. Caldwell S. Russell D. G. (2013). Infection of macrophages with *Mycobacterium tuberculosis* induces global modifications to phagosomal function. Cell. Microbiol. 15, 843–859. 10.1111/cmi.12092 23253353 PMC3620910

[B86] Pol A. Gross S. P. Parton R. G. (2014). Review: biogenesis of the multifunctional lipid droplet: lipids, proteins, and sites. J. Cell Biol. 204, 635–646. 10.1083/jcb.201311051 24590170 PMC3941045

[B87] Puissegur M. P. Botanch C. Duteyrat J. L. Delsol G. Caratero C. Altare F. (2004). An *in vitro* dual model of mycobacterial granulomas to investigate the molecular interactions between mycobacteria and human host cells. Cell. Microbiol. 6, 423–433. 10.1111/j.1462-5822.2004.00371.x 15056213

[B88] Puissegur M. P. Lay G. Gilleron M. Botella L. Nigou J. Marrakchi H.. (2007). Mycobacterial lipomannan induces granuloma macrophage fusion via a TLR2-dependent, ADAM9- and beta1 integrin-mediated pathway. J. Immunol. 178, 3161–3169. 10.4049/jimmunol.178.5.3161 17312164

[B89] Ramakrishnan L. (2012). Revisiting the role of the granuloma in tuberculosis. Nat. Rev. Immunol. 12, 352–366. 10.1038/nri3211 22517424

[B90] Randall P. J. Hsu N. J. Quesniaux V. Ryffel B. Jacobs M. (2015). *Mycobacterium tuberculosis* infection of the 'non-classical immune cell'. Immunol. Cell Biol. 93, 789–795. 10.1038/icb.2015.43 25801479

[B91] Rastogi S. Agarwal P. Krishnan M. Y. (2016). Use of an adipocyte model to study the transcriptional adaptation of *Mycobacterium tuberculosis* to store and degrade host fat. Int. J. Mycobacteriol. 5, 92–98. 10.1016/j.ijmyco.2015.10.003 26927997

[B92] Rhoades E. R. Geisel R. E. Butcher B. A. Mcdonough S. Russell D. G. (2005). Cell wall lipids from *Mycobacterium bovis* BCG are inflammatory when inoculated within a gel matrix: characterization of a new model of the granulomatous response to mycobacterial components. Tuberculosis 85, 159–176. 10.1016/j.tube.2004.10.001 15850754

[B93] Ridley D. S. Ridley M. J. (1987). Rationale for the histological spectrum of tuberculosis. A basis for classification. Pathology 19, 186–192. 10.3109/00313028709077132 3453999

[B94] Russell D. G. (2001). *Mycobacterium tuberculosis*: here today, and here tomorrow. Nat. Rev. Mol. Cell Biol. 2, 569–577. 10.1038/35085034 11483990

[B95] Russell D. G. (2007). Who puts the tubercle in tuberculosis? Nat. Rev. Microbiol. 5, 39–47. 10.1038/nrmicro1538 17160001

[B96] Russell D. G. Cardona P. J. Kim M. J. Allain S. Altare F. (2009a). Foamy macrophages and the progression of the human tuberculosis granuloma. Nat. Immunol. 10, 943–948. 10.1038/ni.1781 19692995 PMC2759071

[B97] Russell D. G. Vanderven B. C. Glennie S. Mwandumba H. Heyderman R. S. (2009b). The macrophage marches on its phagosome: dynamic assays of phagosome function. Nat. Rev. Immunol. 9, 594–600. 10.1038/nri2591 19590530 PMC2776640

[B98] Saka H. A. Valdivia R. (2012). Emerging roles for lipid droplets in immunity and host-pathogen interactions. Annu. Rev. Cell Dev. Biol. 28, 411–437. 10.1146/annurev-cellbio-092910-153958 22578141

[B99] Sassetti C. M. Rubin E. J. (2003). Genetic requirements for mycobacterial survival during infection. Proc. Natl. Acad. Sci. U.S.A. 100, 12989–12994. 10.1073/pnas.2134250100 14569030 PMC240732

[B100] Schaale K. Brandenburg J. Kispert A. Leitges M. Ehlers S. Reiling N. (2013). Wnt6 is expressed in granulomatous lesions of *Mycobacterium tuberculosis*-infected mice and is involved in macrophage differentiation and proliferation. J. Immunol. 191, 5182–5195. 10.4049/jimmunol.1201819 24123681

[B101] Schué M. Maurin D. Dhouib R. N'goma J. C. Delorme V. Lambeau G.. (2010). Two cutinase-like proteins secreted by *Mycobacterium tuberculosis* show very different lipolytic activities reflecting their physiological function. FASEB J. 24, 1893–1903. 10.1096/fj.09-144766 20103719

[B102] Shashkin P. Dragulev B. Ley K. (2005). Macrophage differentiation to foam cells. Curr. Pharm. Des. 11, 3061–3072. 10.2174/1381612054865064 16178764

[B103] Singh V. Jamwal S. Jain R. Verma P. Gokhale R. Rao K. V. (2012). *Mycobacterium tuberculosis*-driven targeted recalibration of macrophage lipid homeostasis promotes the foamy phenotype. Cell Host Microbe 12, 669–681. 10.1016/j.chom.2012.09.012 23159056

[B104] Singh V. Kaur C. Chaudhary V. K. Rao K. V. Chatterjee S. (2015). *M. tuberculosis* secretory protein ESAT-6 induces metabolic flux perturbations to drive foamy macrophage differentiation. Sci. Rep. 5:12906. 10.1038/srep12906 26250836 PMC5388048

[B105] Sirakova T. D. Dubey V. S. Deb C. Daniel J. Korotkova T. A. Abomoelak B.. (2006). Identification of a diacylglycerol acyltransferase gene involved in accumulation of triacylglycerol in *Mycobacterium tuberculosis* under stress. Microbiology 152, 2717–2725. 10.1099/mic.0.28993-0 16946266 PMC1575465

[B106] Tauchi-Sato K. Ozeki S. Houjou T. Taguchi R. Fujimoto T. (2002). The surface of lipid droplets is a phospholipid monolayer with a unique Fatty Acid composition. J. Biol. Chem. 277, 44507–44512. 10.1074/jbc.M207712200 12221100

[B107] Via L. E. Lin P. L. Ray S. M. Carrillo J. Allen S. S. Eum S. Y.. (2008). Tuberculous granulomas are hypoxic in guinea pigs, rabbits, and nonhuman primates. Infect. Immun. 76, 2333–2340. 10.1128/IAI.01515-07 18347040 PMC2423064

[B108] Vilchèze C. Molle V. Carrere-Kremer S. Leiba J. Mourey L. Shenai S.. (2014). Phosphorylation of KasB regulates virulence and acid-fastness in *Mycobacterium tuberculosis*. PLoS Pathog. 10:e1004115. 10.1371/journal.ppat.1004115 24809459 PMC4014462

[B109] Viljoen A. Blaise M. de Chastellier C. Kremer L. (2016). MAB_3551c encodes the primary triacylglycerol synthase involved in lipid accumulation in *Mycobacterium abscessus*. Mol. Microbiol. [Epub ahead of print]. 10.1111/mmi.13482 27513974

[B110] Wältermann M. Steinbüchel A. (2005). Neutral lipid bodies in prokaryotes: recent insights into structure, formation, and relationship to eukaryotic lipid depots. J. Bacteriol. 187, 3607–3619. 10.1128/JB.187.11.3607-3619.2005 15901682 PMC1112053

[B111] Wares F. Balasubramanian R. Mohan A. Sharma S. K. (2005). Tuberculosis control in India. New Delhi: Directorate General of Health Services. Ministry of Health and Family Welfare, in Extrapulmonary Tuberculosis: Management and Control, eds Agarwal S. P. Chauhan L. S. (Publisher Ministry of Health), 95–114.

[B112] WHO (2015). Global Tuberculosis Report. Available online at: http://www.who.int/tb/publications/global_report/en/

[B113] Yang H. Galea A. Sytnyk V. Crossley M. (2012). Controlling the size of lipid droplets: lipid and protein factors. Curr. Opin. Cell Biol. 24, 509–516. 10.1016/j.ceb.2012.05.012 22726586

